# Lifestyle Changes and Colorectal Neoplasia Risk During Colonoscopy Surveillance: A Stage 1 Registered Report

**DOI:** 10.1002/cam4.71440

**Published:** 2026-03-23

**Authors:** Molla M. Wassie, Jean M. Winter, Graeme P. Young, Charles Cock, Maddison Dix, Meseret Derbew Molla, Melkalem Mamuye Azanaw, Norma B. Bulamu, Erin L. Symonds

**Affiliations:** ^1^ College of Medicine and Public Health, Flinders Health and Medical Research Institute Flinders University Adelaide South Australia Australia; ^2^ Department of Gastroenterology Flinders Medical Centre Bedford Park South Australia Australia; ^3^ Department of Biochemistry School of Medicine, College of Medicine and Health Sciences, University of Gondar Gondar Ethiopia

**Keywords:** cohort profile, colorectal cancer, prevention, protocol, registry, risk factors, survey

## Abstract

**Background:**

The development of colorectal neoplasia (precancerous lesions and colorectal cancer [CRC]) is linked to both non‐modifiable and modifiable risk factors among average CRC risk populations. However, no longitudinal studies have assessed non‐modifiable and modifiable risk factors and colorectal neoplasia risk among above‐average risk CRC populations that require regular surveillance colonoscopy. Therefore, this research project will establish a large‐scale colonoscopy surveillance dataset that collects lifestyle and clinical data at multiple time points to assess the effect of lifestyle changes over time on colorectal neoplasia risk.

**Methods:**

A prospective follow‐up study will be conducted among patients who are enrolled in a South Australian colonoscopy surveillance programme. About 21,000 patients will be invited to take self‐reported surveys. Existing programme clinical data will be combined with annual prospectively collected survey data for three consecutive years. Surveys will capture changes in lifestyle patterns over time, including diet, physical activity, sleep quality, smoking habits, and alcohol consumption data at several time points using validated tools. Linear and generalised linear mixed effects models and joint modelling will be used to model continuous, categorical, and a combination of longitudinal and survival data on lifestyle scores and colorectal neoplasia risk, respectively.

**Conclusions:**

This study will be the first to investigate how changes in lifestyle patterns over time influence the development of early precancerous lesions and invasive cancer. Inclusion of high‐risk lifestyle factors when recommending colonoscopy surveillance intervals could provide an improved, personalised CRC prevention strategy to reduce colorectal neoplasia incidence and colonoscopy workload in those at above‐average risk of developing CRC.

## 1 Introduction

Colorectal cancer (CRC) is one of the most common cancers and is the second leading cause of cancer‐related death, with nearly two million new cases and approximately one million deaths every year, worldwide [1]. CRCs take 10–15 years to develop from precancerous adenomas, either through the conventional adenocarcinoma pathway (~65%–85%) or the sessile serrated pathway (~15%) [2]. This provides opportunities for early intervention and cancer prevention by targeting these precancerous lesions before they become invasive [3]. Once individuals are diagnosed with adenomas, they are considered at above‐average risk for future CRC and are recommended to undergo surveillance with colonoscopy every 1, 3, 5, or 10 years, depending on individual risk [4]. International colonoscopy surveillance guidelines are primarily based on adenoma phenotype and degree of family history of CRC, with minimal consideration for other non‐modifiable risk factors, such as lifestyle patterns [5, 6, 7].

CRC is a major public health problem globally, even in developing countries, due to changes in lifestyle patterns, and increasing lifespans due to improvements in survival and affluence [8]. Many non‐modifiable traits are known risk factors for the development of precancerous lesions and CRC such as inherited germline mutations, older age and male sex [9]. However, modifiable risk factors such as low fibre intake, high intake of processed red meat, low calcium intake, physical inactivity, alcohol consumption and cigarette smoking can also contribute up to 70%–75% of the risk for developing precancerous lesions and CRC, significantly more so than non‐modifiable risk factors at only 25%–30% risk [10, 11, 12, 13].

It is widely accepted that diet and lifestyle factors play important roles in the prevention of CRC. This is supported by the Australian National Health and Medical Research Council (NHMRC) and World Health Organisation (WHO) guidelines that recommend above‐average CRC risk individuals should modify their diet and lifestyle to reduce their risk [14, 15]. Research studies on the role of diet in CRC development are mainly limited to assessing individual nutrients (e.g., calcium and vitamin D concentrations) [16] or food types (e.g., red meat) [17], with only single measures of food intake associated with health outcomes, including cancer [18, 19]. There is evidence to show that repeated measurements of food intake at different points in time are better than single measures at one point in time in studying the relationship between diet and disease outcomes [20], and that diet should be assessed as the combination of foods consumed [21, 22]. No studies have investigated dietary and lifestyle patterns over time and their impact on CRC or colorectal neoplasia development in individuals at above‐average risk for CRC.

### 1.1 Study Aims

This study will be conducted in an above‐average risk surveillance cohort and the aims are:

*Aim 1*: To assess diet and lifestyle patterns annually over 3 years.

*Aim 2*: To investigate the association between diet and lifestyle patterns and advanced colorectal neoplasia (advanced adenoma, advanced sessile serrated lesions and/or CRC).

*Aim 3*: To determine if changes in diet and lifestyle patterns over time are associated with the risk of developing colorectal neoplasia.

### 1.2 Proposed Hypotheses

- A combination of poor diet and lifestyle patterns increases the risk for colorectal neoplasia (precancerous lesions and CRC) in an above‐average‐risk population.
- Positive changes that improve diet and lifestyle patterns will reduce the risk of developing colorectal neoplasia.

## 2 Methods

### 2.1 Overview of the Project

The current paper describes a study protocol that aims to assess diet and lifestyle patterns in individuals who are at above‐average risk of CRC. Longitudinal diet and lifestyle data will be collected using self‐administered surveys.

### 2.2 Data Sources and Study Design

**FIGURE 1:**
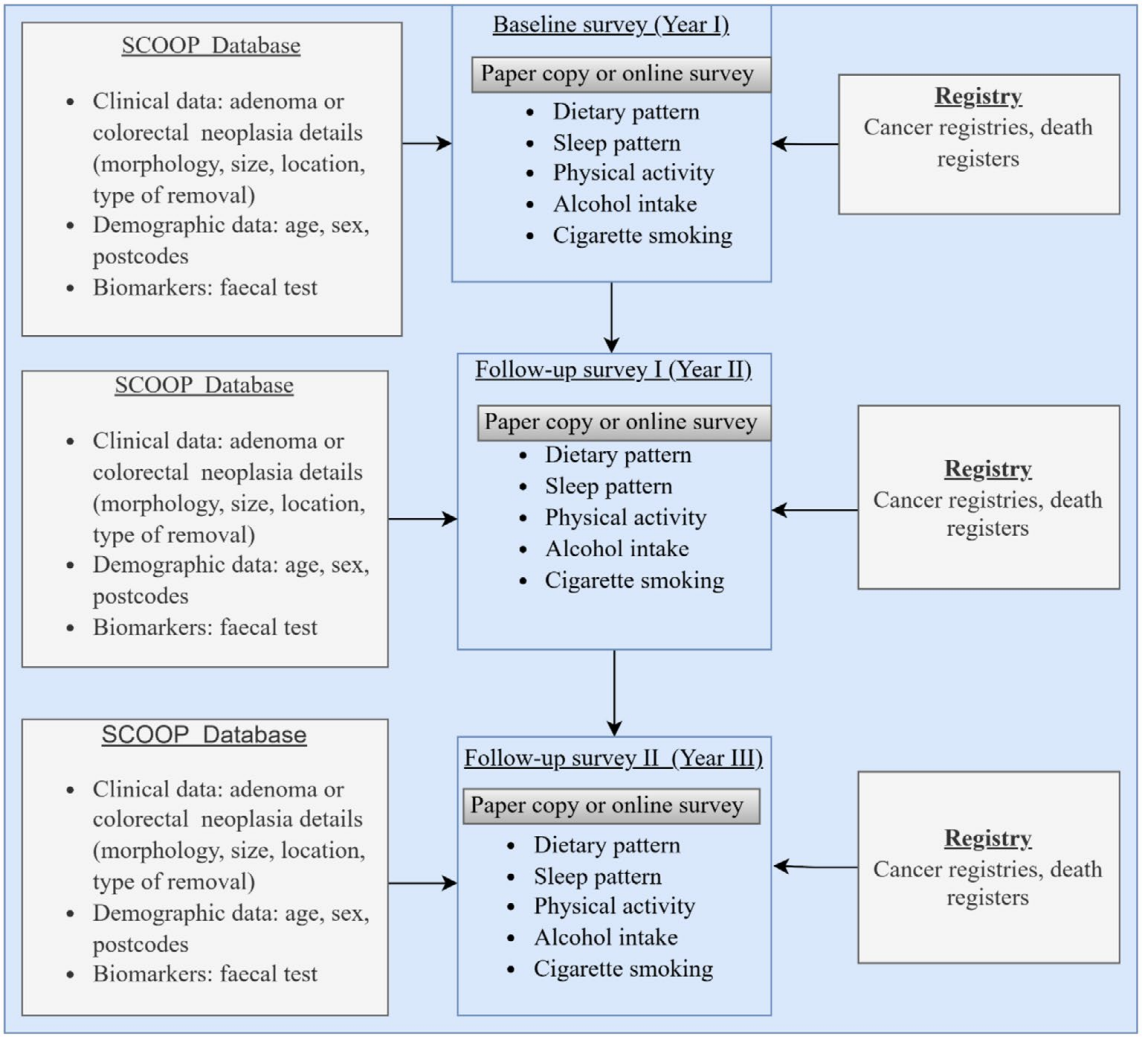
Data linkage workflow for diet and lifestyle patterns over time in individuals undergoing surveillance colonoscopy.

This project is scheduled for completion by October 2026. This is a multicentre study enrolling participants from Flinders Medical Centre, Noarlunga Hospital, and Tennyson Centre Day Hospital in South Australia. The study will also utilise the large Southern Cooperative Program for the Prevention of Colorectal Cancer (SCOOP) database, which manages the Southern Adelaide colonoscopy surveillance programme in South Australia involving over 40,000 patients. This database contains longitudinal clinical data, faecal immunochemical test results, and demographic data, with data collection spanning over 20 years [23]. This resource will be supplemented through linkage to the South Australian cancer and death registers and the collection of additional dietary and lifestyle data via self‐reported survey measures (Figure 1). Clinical records detailing colonoscopy outcomes, including polyp and cancer details (morphology, size, location, type of removal) will be obtained through linkage with the SCOOP clinical database and the South Australian cancer registry.

### 2.3 Study Participants and Eligibility Criteria

All individuals enrolled in the SCOOP programme with an above‐average risk of CRC during the study period will be considered.

#### 2.3.1 Inclusion Criteria

- Over 18 years of age, and
- Enrolled in the SCOOP programme (either private or public) due to:
  ○ A family history of CRC, and/or
  ○ Personal history of adenoma and/or CRC
- Scheduled to undergo regular surveillance colonoscopy.

#### 2.3.2 Exclusion Criteria

- Inability to complete the survey due to language or cognitive issues
- Unable to provide informed consent.

### 2.4 Participant Selection Procedures

To streamline recruitment, participants enrolled in the SCOOP colonoscopy programme who have been actively participating in regular surveillance colonoscopies will be approached for enrolment. A study invitation letter will be sent to eligible participants by mail or email that contains a link to the online survey, participant information sheet, and consent form. Participants will have the opportunity to call the study hotline to request a paper copy of the documents. Each survey is anticipated to take approximately 35–40 min to complete. Survey data will be collected at baseline (year 1) and during two annual follow‐ups (year 2 and 3) (Figure 1).

### 2.5 Data Collection Procedures

#### 2.5.1 Survey Data Collection and Measures

This study has commenced recruitment in November 2023 and data collection will continue up to December 2026. All eligible participants enrolled in the SCOOP programme will be invited to self‐report on the RISC (Assessment of *R*isk factors in *I*ndividuals undergoing *S*urveillance *C*olonoscopy) diet and lifestyle surveys, or the RISC survey in short. First, participants will be given an online or electronic version of the RISC survey to complete. A reminder email or letter will be sent after 4–6 weeks to those who did not complete and return the survey. A paper‐based survey will be sent to participants where the electronic version is not convenient or appropriate. Participant responses will be collected on lifestyle factors, including diet, physical activity, sleep quality, smoking habits, aspirin use, and alcohol intake, using different tools.

The RISC survey includes a combination of the following surveys:

1. *Demographics*: Participants are asked to self‐reporting of the following demographic information: date of birth, sex at birth, current marital status, country of birth, race or ethnicity, highest level of education, current work status, income, household size, and health insurance coverage.
2. *Anthropometry*: Self‐reported height and weight to calculate body mass index (BMI), and waist circumference to calculate central obesity will be collected.
3. *Dietary intake*: A validated food frequency measurement tool from the Australian Eating Survey [24] will be used to collect data on eating behaviours over the past 3 to 6 months. The dietary intake will be collected under the following food category; consumption of ‘milk and dairy foods’, ‘breads and cereals’, ‘sweet and snacks’, ‘main meals’, ‘other foods’ and ‘fruit and vegetables’.
4. *Health‐related quality of life*: The EQ‐5D‐5L will be used to assess participant responses to five dimensions (mobility, personal care, usual activities, pain/discomfort, anxiety/depression)with responses across five levels; no problems, slight problems, moderate problems, severe problems, and extreme problems [24]. The proportion of respondents for each level will be reported and utility scores will generated using the EQ‐5D‐5L crosswalk tariff developed from a general population sample in the United Kingdom [25].
5. *Physical activity*: The International Physical Activity Questionnaire (IPAQ‐L) [26] will be used to ask participants about time spent on physical activity (mild, moderate or vigorous) over the past 7 days, including job‐related activity, transportation, housework, house maintenance, caregiving, recreation, sports, leisure‐time activities, and time spent sitting.
6. *Sleep patterns*: Three validated tools will be implemented: the Pittsburgh Sleep Quality Index (PSQI) [26], the Obstructive Sleep Apnea Screening tool [27] and the Insomnia Severity Index tool [28]. These measures focus on usual sleep habits over the past month, including typical patterns on most days and nights, frequency of sleep disturbances, and the severity of insomnia experienced in the past 2 weeks.
7. *Alcohol intake, smoking habits, or aspirin use*: Survey questions assessing alcohol consumption, smoking habits, and aspirin use were developed by the research team as self‐administered items. These questions were designed to align with Australian guidelines on alcohol and smoking, capturing both current and past behaviours. Additionally, participants were asked whether they use aspirin for the prevention of CRC.

##### 2.5.1.1 Sample Size Determinations

Sample size is determined in STATA software using the Ad Hoc power analysis for the Cox proportional hazards model, assessing the combined effects of diet and lifestyle factors on advanced colorectal neoplasia with the following parameters: confidence level (*α* = 0.05), power (1 − *β* = 80%), hazard ratio (HR = 0.89) [22], and standard deviation (*σ* = 0.05). Based on the above information, the final sample size is 2312. This will be achieved through sending a paper copy or an online survey to SCOOP participants active in the programme (*N* = 21,000), expecting an 11% response rate for the baseline and follow‐up surveys based on previous survey response rates in the SCOOP programme.

##### 2.5.1.2 Study Outcomes

The study has three primary outcomes: (1) Changes in diet and lifestyle patterns over time; (2) Effects of diet and lifestyle behaviours on advanced colorectal neoplasia; and (3) Effects of changes in diet and lifestyle patterns on advanced colorectal neoplasia.

*Advanced colorectal neoplasia* refers to cancer, advanced conventional adenoma (villous features, ≥ 10 mm, ≥ five tubular adenoma, and/or high‐grade dysplasia), advanced sessile serrated lesion (with dysplasia and/or ≥ 10 mm in size) and any size of traditional serrated adenoma.

#### 2.5.2 Data Quality

Appropriate measures will be taken to minimise bias and confounding at all study stages. Both online and paper‐based surveys will be considered to ensure the response rate is not determined by the mode of survey implementation. The online survey administered via REDCap has a pop‐up alert when participants miss responses to questions. Two independent investigators will cross‐check data entry procedures to ensure the quality and integrity of the manually entered data. Multiple imputations will be done to manage missing data.

### 2.6 Proposed Analysis Pipeline

#### 2.6.1 Data Management

Data from the RISC survey will be stored in STATA, Microsoft Access, and Excel on appropriate secure servers. REDCap will be used to collect online survey responses and for the manual entry of data collected from paper‐based copies, which will then be exported to STATA software version 18 for further analysis.

#### 2.6.2 Scoring System

A healthy lifestyle score (0–6) will be derived based on individuals meeting general recommendations from published studies and CRC national guidelines for diet quality, smoking, physical activity, sleeping patterns, weight management and alcohol consumption in which each lifestyle behaviour is allocated a single point on the scale [12].

In addition to the overall lifestyle scores, dietary patterns will be assessed using scores that assess different aspects of a healthy diet:

1. The Mediterranean diet (Mediterranean Diet Score, MDS) [16], and
2. The healthy eating guidelines (Healthy Diet Score, HDS) [28].

As we plan to collect follow‐up surveys and examine the changes overtime, the directional effect of lifestyle changes will be measured in two ways: (1) by evaluating positive or negative fluctuations to the overall score (ranging from 0 to 6), and (2) by analysing changes in the individual scores during the follow‐up period. Changes in both overall and individual scores will be considered.

#### 2.6.3 Statistical Analysis

The analysis method will vary depending on the tested hypothesis. Descriptive statistics will be presented as means with standard deviation and frequencies with percentage for categorised outcomes or scores. Multiple analytical approaches, including linear mixed‐effects models (LMMs), generalised linear mixed‐effects models (GLMMs), and joint modelling of longitudinal and survival analysis, will be applied to each hypothesis using STATA (Statacorp LP, College Station, Texas) and R software (Table 1). LMMs will be used to examine changes in continuous outcomes such as diet score or total healthy lifestyle score over time, accounting for repeated measures. Results will be reported as estimated coefficients (*β*) with a 95% confidence interval (CI) [27]. GLMM will be applied to estimate the change in categorical lifestyle factors over time. Results will be reported as an odds ratio (OR) with 95% CI for GLMM and multivariable logistic regression analysis. Trajectories of change in each diet quality score across the three time points will be analysed using latent class trajectory analysis (LCTA) [29]. Joint modelling of longitudinal and survival analysis (time‐to‐event data) will be utilised to examine the association between change in diet and lifestyle risk factors and the development of advanced neoplasia. The proportional hazard assumption for the survival sub‐model will be checked using the Schoenfeld global test and results will be reported as a hazard ratio (HR) with 95% CI. Stratified analysis will be considered based on colorectal neoplasia sub‐type (e.g., conventional adenoma vs. sessile serrated lesions) and the interval between the prior and latest colonoscopies (e.g., 1 vs. 3 vs. 5 years interval). The analysis will be adjusted for the time from the prior colonoscopy to the baseline or follow‐up survey, and the time between the last survey and the follow‐up colonoscopy.

**TABLE 1:** Overview of hypothesis with corresponding methods of analysis.

Hypothesis	Outcome variable	Covariates	Planned methods of analysis	Assumption
Null hypothesis (*H*_O_): No difference in diet and lifestyle patterns among individuals at above‐average risk of CRC over time Alternative hypothesis (*H*_A_): There are significant variations in diet and lifestyle patterns among individuals at above‐average risk of CRC over time	Change in BMI (mean)	Sex, age, time	Linear mixed model (LML)	Normality Linearity No multicollinearity Constant variance Missing at random
	Change in (categorical data) Smoking status, Alcohol, and physical activity Healthy Lifestyle Score	Sex, age, time	Generalised linear mixed effect models (GLMM)	Missing at random Correct specification of family distribution
	Change in dietary pattern over time Mediterranean Diet Score Healthy Diet Score	Sex, age, time	Principal component analysis Latent class trajectory analysis (LCTA)	Linearity of growth Homoscedasticity Missing at random Sufficient time points (at least three)
*H*_O_: No association between diet and lifestyle behaviours and the development of advanced colorectal neoplasia *H*_A_: There is a significant association between diet and lifestyle factors and developing advanced colorectal neoplasia	Advanced colorectal neoplasia (yes/no)	Lifestyle factors ○ Dietary patterns ○ Sleep pattern ○ Smoking status ○ Physical activity ○ Alcohol intake ○ BMI ○ Healthy Lifestyle Score Confounders: Age, sex, family history, ethnicity, education, marital status, health insurance	Multivariable logistic regressions	Missing at random
*H*_O_: No association between change in lifestyle behaviours and developing advanced colorectal neoplasia *H*_A_: Positive changes that improve diet and lifestyle will reduce the risk of developing advanced colorectal neoplasia	Advanced colorectal neoplasia (yes/no)	Change in lifestyle behaviours Dietary patterns Smoking status Physical activity levels Alcohol intake BMI Healthy Lifestyle score Confounders: Age, sex, family history, ethnicity, education, marital status, health insurance	Joint modelling Longitudinal submodel (LMM or GLMM) and Survival submodel (time‐to‐event‐Cox proportional hazards or parametric survival model)	Assumption for LMM and GLMM

A *p*‐value of less than 0.05 will be considered statistically significant for all planned methods of analysis.

### 2.7 Public Contribution

This study protocol will not involve public contributors in developing and writing the study protocol.

### 2.8 Ethical Considerations

#### 2.8.1 Participant Consent Form

The consent form will outline the brief purpose of a research project, the activities of participants involved in this study (including completing the baseline survey on diet and other lifestyle factors, optionally completing the same survey again 1 and 2 years later and granting permission for the researchers to access the colonoscopy report), the confidentiality, the potential benefits of participating, any possible risks or disadvantages, and the right to withdraw from this project at any time.

#### 2.8.2 Risks or Benefits Related to This Research

There are no foreseeable significant risks or direct benefits to participating in a diet and lifestyle survey. However, participants may feel discomfort while being asked about personal behaviours, including dietary habits, sleeping patterns, smoking, and alcohol consumption and inconvenience from the time taken to complete each survey response. On the other hand, participating in this research will aid researchers in collecting evidence on the effects of diet and lifestyle factors in reducing or increasing the risk of colorectal neoplasia. Summarised data on dietary habits and behavioural scores and standard information about CRC prevention strategies, can be provided after 3 years for those who request it.

#### 2.8.3 Confidentiality

The data collected for this study will be treated as confidential and of a medically sensitive nature. We have procedures in place to maintain the confidentiality of the research data. The project investigators will be the only persons with access to the study deidentified data. Physical copies of consent forms and surveys will be stored in secure, locked cabinets on‐site at the Flinders Centre for Innovation in Cancer and retained for 15 years. All online data will be stored in the SA Health networked databases. Details of the importance of maintaining participants' confidentiality in survey responses have been added to the standardised participant information and consent forms.

#### 2.8.4 Risk Management

If participants develop discomfort when filling the lifestyle behaviours, they will be directed to call their general practitioner (GP), Beyond Blue, or the Cancer Council SA Helpline to arrange counselling or other support. The contact information for Beyond Blue or the Cancer Council SA Helpline is provided in the survey instructions and participant information form. A confidentiality clause has been added to the consent form to help people feel that it is safe to complete and return the survey.

## 3 Discussion

To the best of our knowledge, no study has examined changes in diet and lifestyle patterns among individuals undergoing surveillance colonoscopy in populations with above‐average CRC risk. Previous research has mainly focused on the effects of modifiable lifestyle factors on CRC at specified points in time [30, 31, 32]. Due to a lack of reliable data, it has been challenging to investigate these factors simultaneously over time and understand how their combined influence affects precancerous lesions and CRC development. While such research has been investigated in average‐risk populations, a better understanding of how risk factors interact in influencing colorectal neoplasia development is needed [33, 34].

This research project will be the first in the world to assess the diet and lifestyle patterns of individuals at above‐average risk of CRC over time. The association between diet and lifestyle factors and advanced colorectal neoplasia in an above‐average‐risk population will be assessed. How changes in lifestyle behaviours over time among these individuals are associated with the risk of developing colorectal neoplasia will also be determined. By understanding how diet and lifestyle impact adenoma, sessile serrated lesions, and CRC development in patients at above‐average risk, CRC prevention strategies and messaging can be better targeted with more or less frequent surveillance based on individual risk. This will enable a more personalised surveillance approach, leading to a more efficient use of health resources, reduced cancer incidence, and improved patient outcomes.

The outcomes of this research project will provide an opportunity to more accurately set appropriate colonoscopy surveillance intervals by accounting for currently overlooked lifestyle risk factors, ensuring more personalised CRC prevention strategies. In practice, the provision of colonoscopy services would be more efficient, reducing the stress on the healthcare system and patients. A comprehensive understanding of the interactions between diet and lifestyle behaviours over time, including non‐modifiable risk factors, will serve as a baseline for future investigations into the effectiveness of individualised diet and behaviour interventions on CRC risk. This research project can be translated into clinical practice within surveillance colonoscopy programmes, thereby enhancing CRC prevention in individuals at above‐average risk of CRC.

One of the main strengths of this research project is that survey data will be collected from patients undergoing surveillance colonoscopy in a large, well‐established, evidence‐based colonoscopy surveillance programme (SCOOP) in Australia [23]. Multiple survey data collection time points (three surveys 1 year apart) and inclusions of different lifestyle factors (diet, sleep, physical activity, smoking and alcohol intake) in the survey are other strengths of this study. However, due to the self‐reported survey responses, the study is not free from a social desirability bias.

## Author Contributions

**Molla M. Wassie:** conceptualization (lead), funding acquisition (lead), methodology (lead), project administration (lead), writing – original draft (lead), writing – review and editing (lead). **Jean M. Winter:** conceptualization (equal), project administration (equal), visualisation (equal), writing – original draft (equal), writing – review and editing (equal). **Graeme P. Young:** conceptualization (equal), methodology (supporting), supervision (equal), writing – review and editing (supporting). **Charles Cock:** conceptualization (equal), supervision (equal), writing – review and editing (supporting). **Meseret Derbew Molla:** methodology (supporting), writing – review and editing (supporting). **Melkalem Mamuye Azanaw:** methodology (supporting), writing – original draft (lead), writing – review and editing (supporting). **Maddison Dix:** project administration (equal), writing – review and editing (supporting). **Norma B. Bulamu:** project administration (supporting), supervision (equal), writing – review and editing (equal). **Erin L. Symonds:** conceptualization (lead), funding acquisition (supporting), project administration (lead), visualisation (equal), writing – original draft (equal), writing – review and editing (equal).

## Funding

This project is funded by the National Health and Medical Research Council of Australia (NHMRC) for Dr. Molla Wassie's Emerging Leadership 1 fellowship (2009050).

## Ethics Statement

Ethical approval, number 208.22, has been obtained from the Southern Adelaide Clinical Human Research Ethics Committee (SAC HREC).

## Conflicts of Interest

The authors declare no conflicts of interest.

## Data Availability

The data that support the findings of this study are available on request from the corresponding author. The data are not publicly available due to privacy or ethical restrictions.

## References

[1] F. Bray , M. Laversanne , H. Sung , et al., “Global Cancer Statistics 2022: GLOBOCAN Estimates of Incidence and Mortality Worldwide for 36 Cancers in 185 Countries,” CA: A Cancer Journal for Clinicians 74, no. 3 (2024): 229–263. 38572751 10.3322/caac.21834

[2] L. H. Nguyen , A. Goel , and D. C. Chung , “Pathways of Colorectal Carcinogenesis,” Gastroenterology 158, no. 2 (2020): 291–302. 31622622 10.1053/j.gastro.2019.08.059 PMC6981255

[3] C. Hassan , A. Gimeno‐Garcia , M. Kalager , et al., “Systematic Review With Meta‐Analysis: The Incidence of Advanced Neoplasia After Polypectomy in Patients With and Without Low‐Risk Adenomas,” Alimentary Pharmacology & Therapeutics 39, no. 9 (2014): 905–912. 24593121 10.1111/apt.12682

[4] Surveillance Colonoscopy Guidelines Working Party , Clinical Practice Guidelines for Surveillance Colonoscopy (Cancer Council Australia, 2018), https://wiki.cancer.org.au/australiawiki/index.php?oldid=213462.

[5] Cancer Council Australia Colonoscopy Surveillance Working Party , Clinical Practice Guidelines for the Prevention, Early Detection and Management of Colorectal Cancer: Risk and Screening Based on Family History, ed. C. Bell (Cancer Council Australia, 2024).

[6] S. Gupta , D. Lieberman , J. C. Anderson , et al., “Recommendations for Follow‐Up After Colonoscopy and Polypectomy: A Consensus Update by the US Multi‐Society Task Force on Colorectal Cancer,” American Journal of Gastroenterology 115, no. 3 (2020): 415–434. 32039982 10.14309/ajg.0000000000000544 PMC7393611

[7] M. D. Rutter , J. East , C. J. Rees , et al., “British Society of Gastroenterology/Association of Coloproctology of Great Britain and Ireland/Public Health England Post‐Polypectomy and Post‐Colorectal Cancer Resection Surveillance Guidelines,” Gut 69, no. 2 (2020): 201–223. 31776230 10.1136/gutjnl-2019-319858 PMC6984062

[8] Collaborators GBDCC , “The Global, Regional, and National Burden of Colorectal Cancer and Its Attributable Risk Factors in 195 Countries and Territories, 1990–2017: A Systematic Analysis for the Global Burden of Disease Study 2017,” Lancet Gastroenterology & Hepatology 4, no. 12 (2019): 913–933. 31648977 10.1016/S2468-1253(19)30345-0 PMC7026697

[9] G. Roshandel , F. Ghasemi‐Kebria , and R. Malekzadeh , “Colorectal Cancer: Epidemiology, Risk Factors, and Prevention,” Cancers 16, no. 8 (2024): 1530. 38672612 10.3390/cancers16081530 PMC11049480

[10] R. Sharma , M. Abbasi‐Kangevari , R. Abd‐Rabu , et al., “Global, Regional, and National Burden of Colorectal Cancer and Its Risk Factors, 1990–2019: A Systematic Analysis for the Global Burden of Disease Study 2019,” Lancet Gastroenterology & Hepatology 7, no. 7 (2022): 627–647. 35397795 10.1016/S2468-1253(22)00044-9 PMC9192760

[11] L. A. E. Hughes , C. Simons , P. A. van den Brandt , M. van Engeland , and M. P. Weijenberg , “Lifestyle, Diet, and Colorectal Cancer Risk According to (Epi)genetic Instability: Current Evidence and Future Directions of Molecular Pathological Epidemiology,” Current Colorectal Cancer Reports 13, no. 6 (2017): 455–469. 29249914 10.1007/s11888-017-0395-0 PMC5725509

[12] K. Aleksandrova , T. Pischon , M. Jenab , et al., “Combined Impact of Healthy Lifestyle Factors on Colorectal Cancer: A Large European Cohort Study,” BMC Medicine 12 (2014): 168. 25319089 10.1186/s12916-014-0168-4 PMC4192278

[13] S. K. Veettil , T. Y. Wong , Y. S. Loo , et al., “Role of Diet in Colorectal Cancer Incidence: Umbrella Review of Meta‐Analyses of Prospective Observational Studies,” JAMA Network Open 4, no. 2 (2021): e2037341. 33591366 10.1001/jamanetworkopen.2020.37341 PMC7887658

[14] F. Macrae , T. Lockett , J. Clarke , et al., Clinical Practice Guidelines for the Prevention, Early Detection and Management of Colorectal Cancer, ed. Australia CC (Cancer Council Australia Colorectal Cancer Guidelines Working Party, 2017).

[15] M. Shike , S. J. Winawer , P. H. Greenwald , A. Bloch , M. J. Hill , and S. V. Swaroop , “Primary Prevention of Colorectal Cancer. The WHO Collaborating Centre for the Prevention of Colorectal Cancer,” Bulletin of the World Health Organization 68, no. 3 (1990): 377–385. 2203551 PMC2393072

[16] A. J. Cornish , P. J. Law , M. Timofeeva , et al., “Modifiable Pathways for Colorectal Cancer: A Mendelian Randomisation Analysis,” Lancet Gastroenterology & Hepatology 5, no. 1 (2020): 55–62. 31668584 10.1016/S2468-1253(19)30294-8 PMC7026696

[17] M. Ryan‐Harshman and W. Aldoori , “Diet and Colorectal Cancer: Review of the Evidence,” Canadian Family Physician 53, no. 11 (2007): 1913–1920. 18000268 PMC2231486

[18] W. P. Castelli , “Epidemiology of Coronary Heart Disease: The Framingham Study,” American Journal of Medicine 76, no. 2A (1984): 4–12. 6702862 10.1016/0002-9343(84)90952-5

[19] A. Moskal , H. Freisling , G. Byrnes , et al., “Main Nutrient Patterns and Colorectal Cancer Risk in the European Prospective Investigation Into Cancer and Nutrition Study,” British Journal of Cancer 115, no. 11 (2016): 1430–1440. 27764841 10.1038/bjc.2016.334 PMC5129834

[20] S. A. Bingham , R. Luben , A. Welch , N. Wareham , K. T. Khaw , and N. Day , “Are Imprecise Methods Obscuring a Relation Between Fat and Breast Cancer?,” Lancet 362, no. 9379 (2003): 212–214. 12885485 10.1016/S0140-6736(03)13913-X

[21] B. Magalhães , B. Peleteiro , and N. Lunet , “Dietary Patterns and Colorectal Cancer: Systematic Review and Meta‐Analysis,” European Journal of Cancer Prevention 21, no. 1 (2012): 15–23. 21946864 10.1097/CEJ.0b013e3283472241

[22] P. K. Newby and K. L. Tucker , “Empirically Derived Eating Patterns Using Factor or Cluster Analysis: A Review,” Nutrition Reviews 62, no. 5 (2004): 177–203. 15212319 10.1301/nr.2004.may.177-203

[23] E. L. Symonds , K. Simpson , M. Coats , et al., “A Nurse‐Led Model at Public Academic Hospitals Maintains High Adherence to Colorectal Cancer Surveillance Guidelines,” Medical Journal of Australia 208, no. 11 (2018): 492–496. 29902396 10.5694/mja17.00823

[24] M. Herdman , C. Gudex , A. Lloyd , et al., “Development and Preliminary Testing of the New Five‐Level Version of EQ‐5D (EQ‐5D‐5L),” Quality of Life Research 20, no. 10 (2011): 1727–1736. 21479777 10.1007/s11136-011-9903-x PMC3220807

[25] B. van Hout , M. F. Janssen , Y. S. Feng , et al., “Interim Scoring for the EQ‐5D‐5L: Mapping the EQ‐5D‐5L to EQ‐5D‐3L Value Sets,” Value in Health 15, no. 5 (2012): 708–715. 22867780 10.1016/j.jval.2012.02.008

[26] V. Van Holle , I. De Bourdeaudhuij , B. Deforche , J. Van Cauwenberg , and D. Van Dyck , “Assessment of Physical Activity in Older Belgian Adults: Validity and Reliability of an Adapted Interview Version of the Long International Physical Activity Questionnaire (IPAQ‐L),” BMC Public Health 15 (2015): 433. 25928561 10.1186/s12889-015-1785-3 PMC4427934

[27] L. Zhang , I. Mosquera , E. Lucas , et al., “CanScreen5, a Global Repository for Breast, Cervical and Colorectal Cancer Screening Programs,” Nature Medicine 29, no. 5 (2023): 1135–1145. 10.1038/s41591-023-02315-6 PMC10202799 37106168

[28] M. Maynard , A. R. Ness , L. Abraham , D. Blane , C. Bates , and D. J. Gunnell , “Selecting a Healthy Diet Score: Lessons From a Study of Diet and Health in Early Old Age (The Boyd Orr Cohort),” Public Health Nutrition 8, no. 3 (2005): 321–326. 15918930 10.1079/phn2004679

[29] H. Lennon , S. Kelly , M. Sperrin , et al., “Framework to Construct and Interpret Latent Class Trajectory Modelling,” BMJ Open 8, no. 7 (2018): e020683. 10.1136/bmjopen-2017-020683 PMC6042544 29982203

[30] K. McAloney , H. Graham , C. Law , and L. Platt , “A Scoping Review of Statistical Approaches to the Analysis of Multiple Health‐Related Behaviours,” Preventive Medicine 56, no. 6 (2013): 365–371. 23518213 10.1016/j.ypmed.2013.03.002

[31] L. J. Morris , C. D'Este , K. Sargent‐Cox , and K. J. Anstey , “Concurrent Lifestyle Risk Factors: Clusters and Determinants in an Australian Sample,” Preventive Medicine 84 (2016): 1–5. 26740345 10.1016/j.ypmed.2015.12.009

[32] W. Poortinga , “The Prevalence and Clustering of Four Major Lifestyle Risk Factors in an English Adult Population,” Preventive Medicine 44, no. 2 (2007): 124–128. 17157369 10.1016/j.ypmed.2006.10.006

[33] J. A. Usher‐Smith , F. M. Walter , J. D. Emery , A. K. Win , and S. J. Griffin , “Risk Prediction Models for Colorectal Cancer: A Systematic Review,” Cancer Prevention Research 9, no. 1 (2016): 13–26. 26464100 10.1158/1940-6207.CAPR-15-0274 PMC7610622

[34] Y. Zheng , X. Hua , A. K. Win , et al., “A New Comprehensive Colorectal Cancer Risk Prediction Model Incorporating Family History, Personal Characteristics, and Environmental Factors,” Cancer Epidemiology, Biomarkers & Prevention 29, no. 3 (2020): 549–557. 10.1158/1055-9965.EPI-19-0929 PMC7060114 31932410

